# Etiology, baseline clinical profile and comorbidities of patients with Cushing’s syndrome at a single endocrinological center

**DOI:** 10.1007/s12020-020-02468-1

**Published:** 2020-09-03

**Authors:** Barbara Stachowska, Justyna Kuliczkowska-Płaksej, Marcin Kałużny, Jędrzej Grzegrzółka, Maja Jończyk, Marek Bolanowski

**Affiliations:** Department and Clinic of Endocrinology, Diabetes, and Isotope Therapy, Wroclaw Medical University, Wroclaw, Poland

**Keywords:** Cushing’s syndrome, Hypercortisolism, Hypokalemia, Ectopic ACTH syndrome

## Abstract

**Purpose:**

The aim of this study was to compare phenotype of patients with pituitary, adrenal and ectopic CS and identify the differences regarding biochemical parameters, clinical presentations, and comorbidities in CS patients who were diagnosed at the single endocrinological center in Wroclaw.

**Methods:**

The study population involved 64 patients with CS (53 women and 11 men) diagnosed in Department of Endocrinology, Diabetes and Isotope Therapy in 2000–2018. Patients were divided into three etiologic groups: pituitary dependent-CS (P-CS) (64%), adrenal dependent CS (A-CS) (25%), and CS from an ectopic source (E-CS) (11%).

**Results:**

Percentage of men in the A-CS group was significantly higher than in the other etiologic groups. ACTH, UFC, and cortisol in DST were significantly higher in E-CS group compare to P-CS and A-CS (*p* < 0.05). Mean potassium level in E-CS group was significantly lower than in P-CS and A-CS (*p* < 0.05). Median of time elapsed to diagnosis was significantly lower in the E-CS group compared with either the P-CS and the A-CS group (*p* < 0.01). The most frequently symptoms in CS patients were skin alterations (82.8%), weight gain (81.2%), and hypertension (81.2%).

**Conclusions:**

The epidemiology of CS is changing toward a growing proportion of A-CS. All patients with E-CS presented a profound hypokalemia. Salient hypokalemia could be a biochemical marker more suggestive for E-CS rather than P-CS. The incidence of diabetes is more frequent in E-CS group than in P-CS and A-CS groups.

## Introduction

Endogenous Cushing’s syndrome (CS) is a rare and severe endocrinopathy caused by chronic glucocorticoid excess. Hypercortisolism leads to a characteristic clinical phenotype with components of metabolic disturbances such as: diabetes mellitus (DM), dyslipidemia, central obesity, and hypertension, as well as osteoporosis, muscle weakness, hirsutism, menstrual irregularities, and psychiatric dysfunction [1–10]. The CS incidence is estimated at 0.2–5 per million population per year [11–13]. ACTH-secreting pituitary adenoma is the most common cause of CS and was reported in 66–70% of CS patients [11–13]. Approximately 20–27% of CS cases are caused by primary adrenal disorder—a unilateral adenoma secretion, whereas 5–10%—by ectopic production of ACTH (adrenocorticotropic hormone)/CRH (corticotrophin-releasing hormone) by neuroendocrine tumors [11–13].

Recent publications demonstrated epidemiological, clinical, biochemical features, and diagnostic management during active disease in CS subjects in relation to various CS subtypes [11–13]. Report from a large multicenter study revealed a heterogeneous clinical data of CS depending on gender and etiology. The study involved 481 patients from 57 centers in 26 European countries who were diagnosed in 2000–2010 [12]. ERCUSYN project evaluates the CS prevalence, provides data on diagnostic procedures, clinical features, preoperative and postoperative therapeutic strategies as well as long-term follow-up in CS. The main aim of this registry is to improve CS management and reduce time from first sign or symptom to diagnosis, which may give positive effect in long-term prognosis [12]. One of the reports from ERCUSYN describes clinical presentation, biochemical and hormonal parameters, bone status, comorbidities, and health-related quality of life in 481 patients with CS. Diagnostic management of CS was documented in more recent publications [13].

Cushing’s syndrome is potentially life-threating endocrine disease with high mortality and morbidity. An early diagnosis is very important to reduce the risk of negative long-term outcome. Decrease in exposure to hypercortisolemia may reverse the high mortality and morbidity [14, 15]. The increase in mortality is related to the duration and intensification of excess levels of cortisol. The early diagnosis depends on early suspicion (first clinical signs and symptoms) and then on biochemical confirmation. The clinical presentation of CS in patients with severe overproduction of cortisol is obvious and unmistakable. The most suggestive features that best discriminate CS include: proximal muscle weakness (proximal myopathy), wasting of the extremities with increased fat in the abdomen and face; striae rubra and easy bruising. Many clinical signs and symptoms of CS can occur in the general population and not all patients with CS present with obvious features. The clinical presentation of CS is broad without any leading symptoms, this makes the diagnosis challenging in mild cases or in cyclic disease. Due to variability of the clinical presentation, patients are often referred to specialists because of comorbidities (e.g., osteoporosis) and various complications: cardiovascular (hypertension, pulmonary embolism, stroke, myocardial infarction), neurologic, psychiatric (depression, anxiety), metabolic (hypercholesterolemia, DM), gynecologic (hirsutism, oligomenorrhea, infertility), and dermatologic (acne, facial plethora, striae rubra) [16, 17].

The aim of this study was to compare phenotype of patients with pituitary, adrenal and ectopic CS and identify the differences regarding epidemiological data, biochemical parameters, clinical presentations, and comorbidities in CS patients who were diagnosed at the single endocrinological center in Wroclaw in 2000–2018. In all subjects with CS, both the prevalence of various clinical signs/symptoms and the time to diagnosis in relation to etiology were revealed.

## Materials and methods

The study population involved 64 consecutive patients with CS (53 women and 11 men), diagnosed in the Department of Endocrinology, Diabetes and Isotope Therapy in 2000–2018. We retrospectively studied clinical records of patients from single endocrinological center in Poland. Biochemical and hormonal parameters, comorbidities, histopathology results, and surgical protocols were extracted from medical documentation. The inclusion criteria were as follows: recorded biochemical data compatible with the diagnosis of CS according to the Endocrine Society Clinical Guidelines, the diagnosis of CS ascertained by an endocrinologist at the time of presentation [18]. Patients with diagnosed adrenal cancer were excluded from the study.

The following data were collected by a review of patient medical records: results of basal and dynamic evaluation of the hypothalamic–pituitary–adrenal axis (HPA axis), i.e., urinary free cortisol (UFC), plasma ACTH and cortisol levels—cortisol circadian rhythm (serum cortisol measured at 6.00 a.m., 8.00 a.m., 20.00 p.m., at midnight); lack of normal circadian rhythm of cortisol secretion—midnight cortisol levels >7.5 μg/dL, morning serum cortisol levels after 1 mg dexamethasone suppression test (DST) and suppressibility (with 8 mg dexamethasone). In all subjects, 2–4 24 h UFC measurements were performed. The mean value of 24 h UFC concentrations was calculated and used in further analysis.

According to the Endocrine Society Clinical Guidelines, to confirm the CS diagnosis the following diagnostic criteria were assumed: increased UFC (≥2 tests), serum cortisol levels >1.8 μg/dL after DST, insufficient suppression of serum cortisol during low-dose dexamethasone suppression test – LDDST (≤1.8 μg/dL).

The pituitary etiology of CS was confirmed on the basis of serum cortisol or UFC suppression >50% with the high-dose DST and positive pituitary MRI. Petrosal sinus sampling with CRH stimulation was conducted in ten patients with unclear MRI scan result (lesions <6 mm) or nonconcordant, noninvasive tests and MRI to distinguish between pituitary CS and ectopic ACTH secretion. The etiologic classification of the CS was also based on histologic documentation of the ACTH-secreting pituitary tumor or adrenal tumor.

Histological data were available for 29 pituitary adenomas and in all patients with adrenal tumors. Tissue specimens obtained during surgery of the pituitary gland were collected for a histopathological analysis and immunohistochemical staining. The result of a pathological evaluation was confirmed to be positive if the presence of corticotroph adenoma and resected tumor tissue demonstrated positive ACTH expression. In cases in which histological evidence of corticotroph adenoma was unavailable or uncertain, clinical and biochemical remission after surgery was used to confirm the hypercortisolism.

Subjects were classified on the basis of histologic documentation of ACTH-secreting or adrenal tumor and they were divided into three groups depending on the diagnosis: pituitary-dependent CS (P-CS), adrenal-dependent CS (A-CS)—CS from adrenal adenoma, CS from an ectopic source (E-CS). In patients with E-CS, who did not undergo the removal of the ACTH-secreting tumor, the diagnosis of ectopic source was based on biochemical and radiological results.

The following data were evaluated at diagnosis: gender, anthropometric characteristics: weight (kg), height (cm), body mass index (BMI); CS etiology, the time elapsed from the onset of first signs and symptoms of hypercortisolism to diagnosis; clinical features (moon face, acne, striae rubra, hirsutism, muscle wasting of the extremities, easy bruising, skin atrophy). The prevalence of hypokalemia, hypertension, osteoporosis, fractures, nephrolithiasis, impaired glucose tolerance (IGT), DM and psychiatric disorders was recorded. All the patients with adrenal adenoma of incidental discovery displayed some features of CS: central obesity with supraclavicular fat accumulation, cervical fat pad, muscle atrophy, hirsutism, facial plethora, striae rubra. Height was measured in a standing position with the use of a stadiometer, body weight was measured with the use of a digital electronic scale. BMI was calculated as the weight in kilograms divided by the square of the height in meters. Obesity was diagnosed in subjects with BMI above or equal to 30 kg/m^2^. A BMI between 25 and 30 was considered as an index of overweight [19].

Waist circumference was measured on the waist, in the standing position, midway between the iliac crest and the lower costal margin and cutoff points of 102 cm in men and 88 cm in women were define as abdominal (visceral) obesity [20]. A change in baseline body weight was considered significant when a weight gain was ≥5%.

Blood pressure was measured in the right arm, with the subjects in a relaxed sitting position with a sphygmomanometer. Hypertension was diagnosed when systolic blood pressure (SP) ≥ 140 mm Hg and/or diastolic blood pressure (DP) ≥ 90 mm Hg and/or whether any antihypertensive specific treatment was administrated [21].

Impaired fasting glycaemia (IFG), IGT and DM were diagnosed after an oral glucose tolerance test, DM was diagnosed when fasting blood glucose levels were higher than 126 mg/dl for two consecutive determinations or were 200 mg/dl or greater 2 h after oral glucose and/or whether any antidiabetic medication was administrated. IFG was diagnosed when glucose levels were higher than 100 mg/dl and lower than 125 mg/dl, IGT was diagnosed when blood glucose levels were lower than 126 mg/dl at fasting and 140–200 mg/dl after 2 h a 75 g oral glucose load [22].

Triglycerides (TG), high density lipoprotein (HDL), low density lipoprotein (LDL) and total cholesterol (TC) were measured by standard procedures. Dyslipidemia was diagnosed when TC levels were >200 mg/dL, HDL < 40 mg/dL or TG above >150 mg/dL or whether any specific treatment was given [23].

The adrenocorticotropic hormone (ACTH) levels were measured by chemiluminescence immunoassay method using Immulite 2000 kits (DPC, Germany or USA; Siemens, USA), reference range <46.0 pg/ml. Method sensitivity was 5.0 pg/ml. Cortisol levels were measured by chemiluminescent microparticle immunoassay using Architect i1000SR (Abbott Laboratories, Abbott Park, IL, USA). Reference ranges: before 10 a.m. 3.7–19.4 μg/dL; after 5 p.m. 2.9–17.3 μg/dL. Limit of detection was ≤0.8 μg/dL. Method sensitivity was 1.0 μg/dL. Urine free cortisol (UFC) was measured using a radioimmunoassay method (Immunotech, Beckman Coulter Inc., Prague, Czech Republic), reference range: 14.0–75.0 μg/24 h.

Bone mineral density (BMD) measurements were performed at L1–L4 (the lumbar spine) and femoral neck using the dual-energy X-ray absorptiometry (DXA) method by means of Hologic DPX densitometer. The value of BMD loss was presented as a *T*-score using the following criteria: *T*-score ≥ −1 SD—described as normal; *T*-score between −1 and −2.5 SD—classified as osteopenia; *T*-score < −2.5 SD—classified as osteoporosis. All patients with pituitary-dependent CS underwent pituitary MRI (magnetic resonance imaging).

In case of inconclusive (pituitary tumor lesions <6 mm) or negative MRI scan pictures, inferior petrosal sinus sampling was performed. Pituitary ACTH-tumors were classified as microadenomas (diameter ≤10 mm) or macroadenomas (at least one diameter >10 mm). In case of adrenal adenoma, CT or CT and MRI were performed. Imaging procedures: CT, MRI and octreotide or gallium DOTATATE PET scans were performed to reveal an ectopic ACTH overproduction by a nonpituitary tumor; histopathological data were also collected.

### Statistical analysis

Data were analyzed using the *Prism 5.0 (GraphPad, La Jolla, California, USA) and STATISTICA 10 (StatSoft Inc. Tulsa, Oklahoma, USA)*. Means with standard deviations (SD) and percentages were calculated to describe the clinical characteristics of the patients. The distribution of data was checked with Kolmogorov–Smirnov test. Depending on data distribution, the associations in means between groups were analyzed by Student’s *t* test and ANOVA. Mann–Whitney or Kruskal–Wallis with Dunn’s multiple comparison tests were also used. Proportional differences were tested using the Chi-square (χ^2^) and Fisher’s exact tests. Correlations were determined using Pearson’s test or Spearman’s rank correlation test as appropriate based on variable distribution. *P* values < 0.05 were considered statistically significant.

## Results

### Patient characteristics

In this study, 64 patients were analyzed: 11 men (17%) and 53 women (83%) who were diagnosed in the Department of Endocrinology, Diabetes and Isotope Therapy in 2000–2018. Forty-one of them (64%) had pituitary-dependent CS (P-CS), 16 (25%) adrenal-dependent CS (A-CS), whereas ectopic CS was classified in 7 (11%) patients. Pituitary MRI revealed microadenoma in 26 patients (63%) and macroadenoma in ten cases (24%), including four cases of intrasellar macroadenoma and six cases of extrasellar macroadenoma. In other five patients with P-CS (13%), no specific pituitary abnormal findings were demonstrated. Adrenal dependent CS occurred in 16 patients, with mean adrenal adenoma size of 38 mm, more frequently localized in right adrenal gland. One patient with A-CS had bilateral adenomas. No patient had macronodular adrenal hyperplasia or ACTH-secreting adrenal lesion. Clinical and phenotypic features of all CS patients according to different subtypes of CS are shown in Table 1.

**Table 1:** Baseline characteristics of patients with CS according to different subtypes

	E-CS (*n* = 7)				P-CS (*n* = 41)				A-CS (*n* = 16)				*P* value
	Mean ± SD	Median	*R*	95% CI	Mean ± SD	Median	*R*	95% CI	Mean ± SD	Median	*R*	95% CI	
Time to diagnose (months)	3.4 ± 2.0	3	1–6	(1.6, 5.2)	13.4 ± 8.2	10	4–36	(6, 24)	13.5 ± 7.7	12	1.5–24	(2, 24)	**0.01**
Weight (kg)	74 ± 6	76	68–81	(68, 81)	77 ± 16	74	53–115	(58, 102)	86 ± 15	82	70–116	(72, 102)	NS
Height (cm)	165 ± 5	166	156–170	(160, 170)	164 ± 7	164	150–176	(154, 173)	168 ± 8	168	157–180	(160, 178)	NS
BMI (kg/m^2^)	27.5 ± 2.2	28.5	24.1–29.4	(25.1, 29.4)	28.6 ± 5.7	28.7	19.5–42.1	(21, 37.1)	30.2 ± 3.8	30.5	25.5–36.6	(26.1, 36.1)	NS
Age (years)	54 ± 14	61	32–67	(40, 67)	42 ± 17	39	18–79	(21, 65)	45 ± 13	43	25–67	(31, 66)	NS
SP (<140 mmHg	148 ± 18	150	120–180	(131, 165)	156 ± 13	155	130–180	(140, 180)	153 ± 15	153	120–180	(140, 170)	NS
DP (<90 mmHg)	85 ± 8	80	80–100	(78, 92)	95 ± 8	98	80–112	(90, 100)	93 ± 10	93	60–100	(87, 98)	**0.05**
HOMA	4.3 ± 2	4.3	1.6–6.4	(2.0, 6.4)	3.9 ± 2.2	3.4	1.1–9.9	(3.1, 4.7)	3.5 ± 2	3.9	1–7.4	(2.2, 4.8)	NS
Insulin (µIU/ml) (<29.1 µIU/ml)	13.5 ± 4.7	13	7–21.3	(8.7, 18.4)	16.8 ± 8.9	16	6–44	(13.6, 20)	14.8 ± 9.4	13.6	3.5–38	(8.8, 20.8)	NS
Glucose mg/dl (65–99 mg/dl)	132 ± 42	121	87–179	(93, 171)	93 ± 19	89	70–145	(74, 116)	97 ± 19	91	77–140	(79, 130)	**0.05**
TG mg/dL (<150 mg/dL)	126 ± 63	115	48–223	(60, 191)	141 ± 55	136	64–312	(123, 158)	169 ± 89	131	73–400	(120, 219)	NS
LDL mg/dL (<115 mg/dL)	100 ± 52	88	50–195	(45, 154)	120 ± 36	119	61–226	(109, 132)	134 ± 30	136	79–181	(117, 152)	NS
UFC μg/24h (14.0–75.0 μg/24 h)	2770 ± 1719	2496	1150–5800	(1180, 4360)	463 ± 335	367	85–1276	(355, 570)	419 ± 415	211	78–1624	(189, 649)	**0.01**
CDST µg/dL (<1.8 µg/dL)	58.9 ± 49.7	40.1	16–157	(12.8, 104.8)	22.1 ± 45.8	14.5	4.6–306	(7.6, 36)	14.6 ± 11.6	5	5.9–55.1	(6.3, 20)	**0.01**
ACTH pg/ml (<46.0 pg/ml)	238 ± 124	273	92–425	(123, 352)	73 ± 43	63	25–212	(60, 87)	6 ± 3	5	5–15	(5, 8)	**0.01**
CDST8 P µg/dL	95.1 ± 69	79.4	35–171	(−77.1, 267.4	7.5 ± 9.1	5.2	3–39	(4.4, 11.5)	10.2 ± 4.5	10	2.2–17.3	(7.2, 13.3)	**0.01**
CDST8 U μg/24h	3311 ± 1948	2614	2614–7271	(2210, 8411)	222 ± 561	30	4–2760	(−10, 453)	279 ± 429	128	3–1462	(−8.4, 568)	**0.01**
K (3.5–5.0 mEq/L)	2.9 ± 0.7	2.9	2–4	(2.2, 3.5)	3.7 ± 1.3	4.1	2.9–5	(3, 4.6)	4.0 ± 0.6	4.2	3.1–5	(3.7, 4.3)	**0.01**

*P-CS* pituitary dependent CS, *A-CS* adrenal dependent CS from adrenal adenoma, *E-CS* CS from an ectopic source, *R* range, *BMI* body mass index, *CS* Cushing’s syndrome, *DM* diabetes mellitus, *CDST* cortisol level after overnight 1 mg dexamethasone suppression test, *IFG* impaired fasting glucose, *UFC* urinary free cortisol, *SP* systolic blood pressure, *DP* diastolic blood pressure, *CDST8 P* cortisol in dexamethasone suppression test 8 mg plasma, *CDST8 U* cortisol in dexamethasone suppression test 8 mg urine, *K* Potassium

Bold values indicate statistically significant *p* < 0.05

Women were more likely to have pituitary ACTH-secreting adenoma than men, with a ratio of 5:1. The percentage of men in A-CS group was significantly higher than in P-CS group (no diagnosed men in E-CS group). Ectopic CS was detected in seven patients: bronchial neuroendocrine tumors (one atypical) in two patients, pancreatic neuroendocrine tumor in two patients, small intestine neuroendocrine tumor in one patient, and thymic carcinoid in one patient. In one case, the localization of ectopic ACTH secretion was not revealed despite a profound investigation.

In the present study, mean age at diagnosis was 44 ± 17 years (range: 18–79 years) in women and 43 ± 10 years (range: 25–67 years) in men. There was no significant difference in age between women and men. No significant differences among P-CS, A-CS and E-SC groups were observed regarding age, weight, and BMI. Mean BMI was 28 ± 5 kg/m^2^ (range: 19–42) in women and 31 ± 5 kg/m^2^ (range: 22–37) in men (*p* = ns). Mean weight in men was significantly higher (*p* < 0.05)—91 ± 17 kg (range 61–116) than in women—76 ± 13 kg (range: 53–108). Women had higher ACTH, UFC, and serum cortisol levels at the time of diagnosis compared to men. The clinical characteristics and biochemical features of men and women are shown in Table 2.

**Table 2:** Baseline characteristics of the patients with CS according to gender

	Women (53)				Men (11)				*P* value
	Mean ± SD	Median	*R*	95% CI	Mean ± SD	Median	*R*	95% CI	
Time to diagnose (months)	11.6 ± 7.6	9.0	1.0–36.0	(9.5, 13.7)	15.3 ± 9.9	11.5	2.0–36.0	(9.0, 21.6)	NS
Weight (kg)	76 ± 13	73	53–108	(71, 80)	90 ± 17	87	61–116	(78, 103)	**0.01**
Height (cm)	164 ± 6	164	150–178	(161, 165)	171 ± 6	173	157–180	(169, 176)	NS
BMI (kg/m2)	28.5 ± 5.0	28.7	19.5–42.1	(26.9, 30.0)	30.7 ± 5.1	30.1	21.9–37.1	(27.0, 34.3)	NS
Age (years)	44 ± 17	43	18–79	(39, 47)	43.36 ± 10.13	40	25–67	(38, 52)	NS
SP (mmHg) (<140 mmHg	153 ± 14	155	120–180	(149, 157)	160 ± 14.7	155	140–180	(150, 169)	NS
DP (mmHg) (<90 mmHg)	93 ± 9.4	95	60–112	(90, 96)	95 ± 4	95	90–100	(92, 98)	NS
Glucose (mg/dl) (65–99 mg/dl)	98 ± 26	91	70–179	(91, 106)	99 ± 23	93	74–140	(84, 113)	NS
Insulin (µIU/ml) (<29.1 µIU/ml)	12 ± 9.8	12.0	4.0–44.4	(9.7, 15.1)	15.0 ± 10.6	16.5	6.4–38.0	(8.2, 21.7)	NS
HOMA	3.0 ± 2.5	2.7	2.0–9.9	(2.3, 3.7)	3.7 ± 2.5	4.6	2.9–7.4	(2.2, 5.3)	NS
TG mg/dL (<150 mg/dL)	138 ± 73	132	70–400	(117, 158)	149 ± 59	135	72–279	(112, 187)	NS
LDL mg/dL (<115 mg/dL)	110 ± 49	110.0	80–226	(97, 124)	131 ± 30	134	72–181	(112, 151)	NS
UFC μg/24h (14.0–75.0 μg/24 h)	759 ± 1050.3	457	12–5800	(467, 1052)	468 ± 347	240	150–1136	(235, 702)	**0.05**
CDST µg/dL (<1.8 µg/dL)	30 ± 54.1	15.6	4.6–306.0	(15.4, 45.3)	21.6 ± 25.5	13.2	7.2–101.0	(5.4, 37.8)	**0.05**
ACTH pg/ml (<46.0 pg/ml.	77 ± 85	57	5–425	(53, 100)	65 ± 71	59	5–212	(17, 113)	**0.05**
CDST8 P µg/dL	16 ± 32	7.0	5–171	(4.0, 27.8)	14.9 ± 13.2	11.1	2.2–39.0	(1.1, 28.7)	NS
CDST8 U μg/24 h	822 ± 1830	100	2–7271	(183, 1460)	275 ± 526	100	3–1462	(−210, 762)	**0.05**
K mEq/L (3.5–5.0 mEq/L)	4.0 ± 0.7	4.1	2.0–5.0	(3.8, 4.2)	3.9 ± 0.5	4.1	3.0–4.5	(3.5, 4.3)	NS

*P-CS* pituitary dependent CS, *A-CS* adrenal dependent from adrenal adenoma CS, *E-CS* CS from an ectopic source, *BMI* body mass index, *CS* Cushing’s syndrome, *DM* diabetes mellitus, *CDST* cortisol level after overnight 1 mg dexamethasone suppression test, *IFG* impaired fasting glucose, *UFC* urinary free cortisol, *SP* systolic blood pressure, *DP* diastolic blood pressure, *CDST8 P* cortisol in dexamethasone suppression test 8 mg plasma, *C DST8 U* cortisol in dexamethasone suppression test 8 mg urine, *K* Potassium

Bold values indicate statistically significant *p* < 0.05

**Fig. 1:**
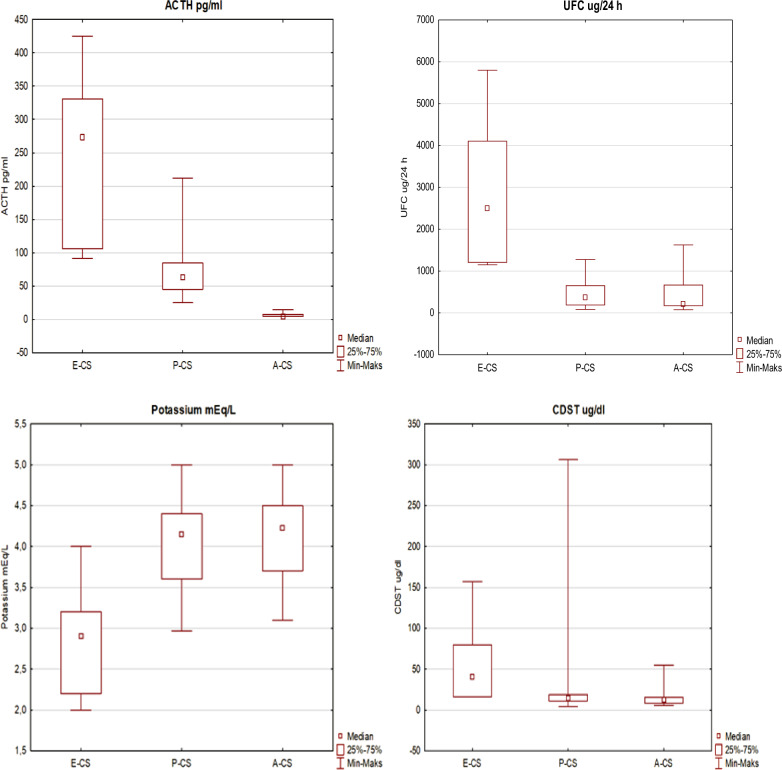
UFC, ACTH, Potassium and cortisol values after 1 mg DST in each etiologic group of CS. P-CS pituitary dependent CS, A-CS adrenal dependent from adrenal adenoma CS, E-CS CS from an ectopic source, CS Cushing’s syndrome, CDST cortisol level after overnight 1 mg dexamethasone suppression test, UFC urinary free cortisol

Mean glucose level in E-CS group was 132 ± 42 mg% (range: 87–179) and was significantly higher than in P-CS (mean: 93 ± 19 mg%; range: 70–145) and A-CS group (mean: 97 ± 19 mg%; range: 77–140). Mean potassium level in E-CS group (2.9 ± 0.7 mEq/L; range: 2.0–4.0) was significantly lower than in P-CS and A-CS (*p* < 0.01). ACTH, UFC, and cortisol in DST were significantly higher in E-CS group compared to P-CS and A-CS (*p* < 0.01) groups (Fig. 1). Plasma and urine cortisol in LDDST was also significantly higher in E-CS group compared to P-CS and A-CS patients (*p* < 0.01 for both comparisons).

We found no significant correlation between age at diagnosis and the levels of UFC, cortisol, and ACTH plasma. ACTH concentrations were correlated with urine, serum cortisol, and potassium levels (*p* < 0.005). No correlation was detected between ACTH, the time to diagnosis and age.

**Fig. 2:**
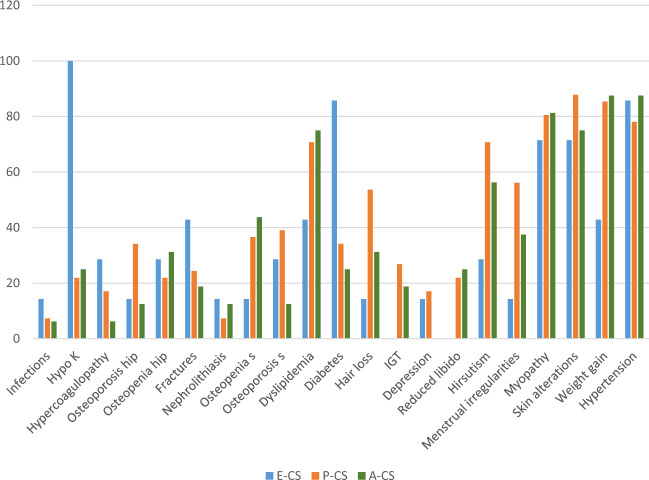
Distribution of symptoms (%) in the overall patients with CS. P-CS pituitary dependent CS, A-CS adrenal dependent CS from adrenal adenoma, E-CS CS from an ectopic source, IGT impaired glucose tolerance

**Fig. 3:**
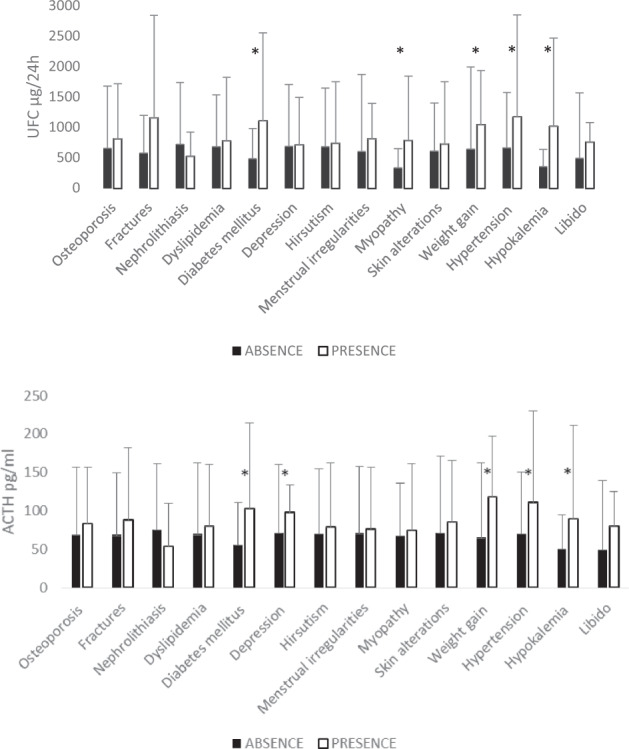
Mean UFC, ACTH, and CDST according to the presence or absence of signs or symptoms in patients with CS. *indicates statistical significance. UFC urinary free cortisol. CDST cortisol in dexamethasone suppression test 1 mg plasma

Median time elapsed between the onset of first (Fig. 2 and 3) symptoms of hypercortisolism and the final CS diagnosis was similar in women—9.0 months (range: 1–36 months) and men—11.5 months (range: 2–36 months). In all cases, negative correlation was found among UFC, ACTH, and the time to diagnosis (*p* = 0.05). In P-CS group, the median time from first symptoms of cortisol excess to diagnosis was 10 months (range: 4–36), whereas in A-CS and E-CS groups—12 months (range: 1.5–24 months) and 3 months respectively. Median time elapsed between the onset of symptoms and the time of diagnosis was significantly lower in E-CS group compared to other two groups (*p* < 0.01). Comorbidities and symptoms of CS in the overall population of patients with CS and each etiologic group are described in Table 3.

**Table 3:** Clinical characteristics of patients with CS and each etiologic group. Data for each etiologic group and the overall series are expressed as number and percentages of patients with a sign or symptom

No of patients	64		7		41		16		*P* value
	Overall	%	E-CS	%	P-CS	%	A-CS	%	
Infections	5	7.8	1	14.3	3	7.3	1	6.2	NS
Hypokalemia	20	31.2	7	100.0	9	21.9	4	25.0	**0.01**
Hypercoagulopathy	10	15.6	2	28.6	7	17.1	1	6.2	**0.05**
Osteoporosis hip	17	26.6	1	14.3	14	34.1	2	12.5	NS
Osteopenia hip	16	25.0	2	28.6	9	21.9	5	31.2	NS
Fractures	16	25.0	3	42.9	10	24.4	3	18.7	NS
Nephrolithiasis	6	9.4	1	14.3	3	7.3	2	12.5	NS
Osteopenia s	23	35.9	1	14.3	15	36.6	7	43.7	NS
Osteoporosis s	20	31.2	2	28.6	16	39.0	2	12.5	NS
Dyslipidemia	44	68.7	3	42.9	29	70.7	12	75.0	**0.05**
Diabetes mellitus	24	37.5	6	85.7	14	34.1	4	25.0	**0.01**
Hair loss	28	43.7	1	14.3	22	53.7	5	31.2	**0.05**
IGT	14	21.9	0	0.0	11	26.8	3	18.7	NS
Depression	8	12.5	1	14.3	7	17.1	0	0.0	NS
Reduced libido	13	20.3	0	0.00	9	21.9	4	25.0	NS
Hirsutism	40	62.5	2	28.6	29	70.7	9	56.2	**0.05**
Menstrual irregularities	30	46.9	1	14.3	23	56.1	6	37.5	**0.05**
Myopathy	50	78.1	5	71.4	33	80.5	13	81.2	NS
Skin alterations	53	82.8	5	71.4	36	87.8	12	75.0	NS
Weight gain	52	81.2	3	42.9	35	85.4	14	87.5	**0.05**
Hypertension	52	81.2	6	85.7	32	78.0	14	87.5	NS

*P-CS* pituitary dependent CS, *A-CS* adrenal dependent CS from adrenal adenoma, *E-CS* CS from an ectopic source, *IGT* impaired glucose tolerance

Bold values indicate statistically significant *p* < 0.05

Clinical data of all patients revealed the most common signs and symptoms at diagnosis: skin alterations (53/64–82.8%), weight gain and hypertension (52/64–81.2%). Hypertension was the most frequent clinical feature in patients with E-CS (85.7%) and A-CS (87.5%). Weight gain (87.8%) and skin alterations (85.4%) were the most frequent clinical findings in P-CS group. Hirsutism occurred in 70.7% of women (premenopausal and postmenopausal) in P-CS group and this percentage was significantly higher compared to both E-CS (28.6%) and A-CS (56.2%) groups. Menstrual irregularities were found in 56.1% of premenopausal women with P-CS and only in 14.3% of women with E-CS. The incidence of weight gain was significantly higher in P-CS group (85.4%) and A-CS group (87.5%) compared to E-CS group (*p* < 0.01 for both comparisons). Obesity defined by BMI was diagnosed in 31% (*n* = 20/64) of subjects, 94% of patients were overweight. Abdominal (visceral) obesity was defined in 92% patients (*n* = 59/64).

Diabetes mellitus was found in 85.7% of patients with E-CS and this prevalence was significantly higher compared to that in the other groups (34.1%—P-CS group and 25%—A-CS group; *p* < 0.01 for both comparisons). A family history of DM was present in 46% (*n* = 11/24) of patients with DM and 55% (*n* = 22/40) of subjects without DM. There were no significant differences in prevalence of DM between patients with a family history of DM and those without family history of DM.

Skin alterations (85%), myopathy (85%), and weight gain (83%) were the most prevalent signs and symptoms at diagnosis in women. Hypertension (91%), myopathy (73%), weight gain (73%), and reduced libido (46%) were more frequent at time of diagnosis in men than in women (45% vs. 25%, *p* < 0.05).

Osteopenia of the spine was reported in 43.7% of patients in A-CS group, 36.5% of patients in P-CS group, and 14.3% of patients in E-CS group. Osteopenia of the hip occurred in 28.6% of patients in E-CS group, 21.9% of patients in P-CS group, and 31.2% of patients in A-CS group. The prevalence of osteoporosis of the spine was higher in P-CS group (39.0%) compared to E-CS (28.6%) and A-CS (12.5%) groups; however, this did not reach statistical significance. The prevalence of osteoporosis of the hip was significantly higher in women compared to men (28.0% vs. 18.2%, *p* < 0.05). Osteoporosis of the spine was reported in 32.1% of women and 27.3% of men and was significantly more prevalent in cases with vertebral fractures compared to those with normal *T*-score (*p* < 0.05). Vertebral fractures were documented in 12 patients with osteoporosis and in four patients with osteopenia at any site. Patients with ectopic source of CS had more fractures (mainly vertebral) at the time of diagnosis compared to P-CS and A-CS groups (42.7% vs. 24.4% and 18.7%, respectively); however, this difference did not reach statistical significance. Characteristics of bone mineral status in each etiologic group of CS are shown in Table 4.

**Table 4:** Prevalence of osteoporosis and fractures in all patients with CS each etiologic group

	64		7		41		16	
	Overall	%	E-CS	%	P-CS	%	A-CS	%
DXA osteoporosis hip	17	26.6	1	14.3	14	34.1	2	12.5
DXA osteopenia hip	16	25.0	2	28.6	9	21.9	5	31.2
DXA osteopenia spine	23	35.9	1	14.3	15	36.6	7	43.7
DXA osteoporosis spine	20	31.2	2	28.6	16	39.0	2	12.5
Fractures	16	25.0	3	42.9	10	24.4	3	18.7

*P-CS* pituitary dependent CS, *A-CS* adrenal dependent CS from adrenal adenoma, *E-CS* CS from an ectopic source, *DXA* dual energy X-ray absorptiometry

## Discussion

In our monocentric retrospective study we compared clinical features, biochemical, and hormonal parameters as well as bone mineral status of 64 CS patients with different etiologies, who were diagnosed in one endocrinological center in Poland. Pituitary adenomas (P-CS) were documented in 64% of cases, adrenal adenomas in 25% of cases, whereas CS from an ectopic source in 11% of all CS patients. Data regarding the prevalence of P-CS and E-CS are consistent with previous reports [24–27]. In recent publications, adrenal adenomas were documented in 5–22.3% of CS cases [25–28]. In cohort study from Israel, the incidence of adrenal etiology of CS was 38.8% (including the patients with adrenal carcinoma-AC) and was higher compared to our data (we excluded patients with AC) [29]. According to the study from Israel, a growing proportion of diagnosed A-CS relative to P-CS might be attributed to better detection of adrenal mass—incidentalomas [29]. Adrenal lesions are often detected unexpectedly by means of an increased use and improved imaging examinations performed for reasons unrelated to any suspicion of adrenal disease. Available guidelines for the management of incidentaloma recommended an accurate assessment for every patient with an adrenal mass larger than 10 mm, including thorough clinical examination for signs and symptoms of adrenal hormone excess, and an adequate hormonal evaluation including cortisol levels [30, 31]. According to this consensus, the rising prevalence of incidentally detected adrenal masses results in increasing disclosure of A-CS.

There was no significant difference in the age at diagnosis in each group (P-CS, E-CS, and A-CS). These data are consistent with previous reports [26]. However, according to ERCUSYN database, patients with adrenal adenoma were significantly older than those with P-CS [12]. In the study from Israel, mean age at diagnosis of pituitary CS was 42.2 years but the mean age of patients with adrenal CS was 51.6 years [29]. Polish patients with A-CS were older compared to P-CS and E-CS cases—mean age at diagnosis was 45.25 years in A-CS group vs. 41.88 years in P-CS group. Previous studies confirmed that incidence of adrenal incidentalomas increases with age [31].

Our analysis revealed that in all patients with CS the most common clinical findings at diagnosis were: skin alterations (82.8%), weight gain (81.2%), and hypertension (81.2%). Weight gain (in 81% of CS cases) was the most prevalent sign in ERCUSYN database, hypertension was documented in 78% of CS cases and skin alterations in 73% of all CS cases [12]. Skin alterations (87.8%) were the most common clinical findings in our P-CS group, hypertension (85.7%) in E-CS group, and weight gain and hypertension (87.5%) in A-CS group. According to ERCUSYN data, the most common clinical features were: weight gain in P-CS group (82%), hirsutism (92%) in E-CS group, weight gain and hypertension (82%) in A-CS group [12]. The prevalence of CS signs and symptoms in our study was not different from that described in other studies—skin alterations, weight gain and hypertension were the most common clinical features of CS at diagnosis [7, 8, 32]. In a univariate analysis from prospective multicenter study, Leon-Justel et al. indicated a risk score for the identification of CS. The authors concluded that osteoporosis, muscle atrophy, and dorsocervical fat pad were significantly associated with CS [33].

There were no differences in the mean BMI, age, and weight among patients in P-CS, A-CS, and E-CS groups. In ERCUSYN database, the incidence of weight gain in E-CS group was lower than in our study (42.9% vs. 70%) [12]. Muscle weakness was more common in our cases (78.1%) than in others (56–60%) [7, 32]. According to ERCUSYN database, myopathy occurred in 73% cases [12]. Clinical manifestations of CS in men included reduced libido and impotence. According to our data, the prevalence of low libido was significantly higher in men than in women. In women, the more common than low libido were menstrual irregularities and hyperandrogenism. The prevalence of depression and reduced libido differed between our data and two other studies, i.e., such clinical findings were less common in our groups [7, 12]. Interestingly, depression prevalence was associated with increased ACTH secretion and was significantly higher in patients with P-CS compared to A-CS group. Our data are consistent with other studies, where ACTH was associated with psychiatric disorders and cognitive impairment [8]. These findings may result in a better understanding of the pathophysiological role of ACTH associated with psychiatric disorders. However, Sonino et al. proved no significant differences in the prevalence of depression between P-CS and A-CS as well as showed that depression in CS was significantly associated with 24-UFC, female sex, age, and various severe comorbidities [9].

Arterial hypertension may be the first symptom of glucocorticoid excess. Previous studies confirmed the prevalence of hypertension in 55–85% of patients with CS [1]. In a retrospective study, Torpy et al. documented hypertension in 45 (78%) out of 58 ectopic CS patients [34]. Two recent studies reported CS prevalence in 0.5–1% of all patients with secondary hypertension [35, 36]. Hypertension was more prevalent in patients with A-CS than those with E-CS and P-CS (87.5% vs. 85.1% and 78.0%, respectively), but the difference did not reach statistical significance. In ERCUSYN database, hypertension was the most frequent in ectopic CS (88%) [12].

Diabetes mellitus is one of predominant comorbidities in CS and it depends on a duration and a degree of hypercortisolism. Our analysis has shown the variable prevalence of DM in each etiologic group. DM was more frequently documented in patients with ectopic CS compared to those with P-CS and A-CS (85.7% vs. 34.1% and 25.0%, respectively). This data is consistent with two other studies on patients with ectopic CS; DM was described in 75–80% of cases [37, 38]. According to ERCUSYN registry, an increased DM prevalence (74%) occurred in ectopic CS, as compared to pituitary (33%) and adrenal CS (34%) [12]. Nevertheless, recent large studies documented a lower DM prevalence in patients with ectopic etiology [27, 39]. Ilias et al. recorded diabetes in 50% patients with ectopic CS and Isidori et al. documented 37.5% prevalence in a series of 40 patients [27, 39]. ERCUSYN registry revealed that patients with ectopic ACTH-secreting tumor were more often consulted and diagnosed by a diabetologist than those with other CS etiologies [12]. The main difference between the two forms of ACTH-excess is a more severe degree of hypercortisolism in the ectopic CS.

Hypercortisolemia results in increased gluconeogenesis, altered insulin secretion, increased insulin resistance and the presence of DM and metabolic complications [40]. The severity of cortisol excess is related to abnormal glucose metabolism. The link between impaired glucose metabolism, DM, and CS has been revealed in various studies [41]. Catargi et al. documented a 2% prevalence of CS after screening 200 diabetic patients with HbA1c >8% and BMI >25 kg/m^2^ [42]. Leibowitz et al. found three cases of CS among 90 diabetic patients (3.3%) with HbA1c >9% and BMI >25 kg/m^2^ [43]. Chiodini et al. revealed the presence of subclinical CS in 9.4% of diabetic patients and in 2.1% of patients from the control groups [44]. By contrast, Reimondo et al. reported only one case of subclinical CS among 100 newly diagnosed diabetic patients [45]. Newsome et al. screened 171 overweight diabetic patients and did not detect any CS cases [46]. Recent systematic review and meta-analysis on the prevalence of hypercortisolism in diabetic patients have recorded CS in 3% of diabetic study groups [47]. In a cohort study, out of 813 patients with DM, Terzolo et al. observed the presence of previously unsuspected CS in 0.7% subjects (the study included unselected population, i.e., not only patients with signs and symptoms indicating a high probability of hypercortisolism). The incidence of definitive CS (after the use of a more specific cutoff point for 1 mg DST at 5.0 ug/dl) was 5.1% among patients with HbA1c >9.0%, despite intensive treatment and blood pressure not on target despite the administration of three drugs [48]. These data may be a suggestion for physicians to identify other features typical for CS in patients with uncontrolled DM despite appropriate treatment. Poorly controlled hyperglycemia and weight gain may require investigation and may lead to CS diagnosis.

It is worth to mention that other non-modifiable risk factors, such as age, genetic predisposition, and modifiable risk factors as lifestyle, which are independent of hypercortisolism strongly contribute to the impairment of glucose tolerance in patients with CS [49]. Giardano C. et al. reported that familial history appears of DM predisposed CS patients to worse metabolic abnormalities [49]. Authors suggested that DM is not a simple consequence of hypercortisolism and that both family history—genetic predisposition of DM and hypercortisolism (duration of disease, degree of hypercortisolism) together may lead to impaired glucose metabolism [49].

Dyslipidemia occurred more often in A-CS and P-CS groups than in E-CS group (75% and 70% vs. 42%, respectively). It should also be noted that the most common complications of hypercortisolemia in E-CS group was hypokalemia (100% incidence) and in most of them profound hypokalemia occurred, which is consistent with previous studies [1, 2]. Hypokalemia was present in 21.9% of patients with P-CS and in 25.0% of patients with A-CS. In other studies hypokalemia was much more prevalent in E-CS group than in patients with other causes of CS, affecting 33 out of 58 patients (57%) [34]. In our study, the prevalence of hypokalemia was significantly correlated with serum cortisol, ACTH, and 24-UFC levels.

Cushing’s syndrome is a rare endocrinopathy and the lack of typical signs and symptoms makes it difficult to diagnose the disease on time. We documented shorter average time to diagnosis in patients with ectopic ACTH-secreting tumors compared to those in P-CS and A-CS groups. These data are consistent with previous reports that revealed a more aggressive and rapid onset of clinical signs and symptoms in cases with ectopic CS source [7, 11].

In our study, the time elapsed between the onset of first symptoms of hypercortisolism and the diagnosis of CS was significantly shorter in patients with ectopic ACTH secretion, in whom the highest UFC and cortisol serum levels were recorded. The levels of UFC, cortisol serum, and ACTH were significantly higher in the ectopic group as compared to patients in P-CS and A-CS groups. According to ERCUSYN registry, the median time from the onset of first symptoms to diagnosis was 0.5 year in the ectopic group—these data are similar to the results of our study [12]. In meta-analysis involving 44 studies, Rubinstein G. et al. reported that the average time to diagnosis in all cases of CS constituted 34 months; in ectopic CS the time was significantly shorter (14 months) than in adrenal (30 months) and pituitary CS (38 months) [50].

Our E-CS patients had various metabolic complications due to hypercortisolemia, including hypertension (85%), DM (85%), and dyslipidemia (42%). The DM incidence was significantly higher in E-CS group compared to P-CS and A-CS groups. Cortisol overproduction due to ectopic aggressive ACTH-secreting tumors lead to more severe clinical symptoms (DM, hypertension, profound hypokalemia), their much more rapid onset as well as the presence of life-threatening comorbidities. In our study, the most prevalent signs were: skin lesions (87.8%) in P-CS, hypertension (85.7%) in E-CS, weight gain and hypertension (87.5%) in A-CS group. In ERCUSYN data, the most common symptoms were: weight gain in P-CS (82%), hirsutism in E-CS (92%), weight gain and hypertension in A-CS (82%) [12]. We found no difference in the severity of cortisoluria between patients with P-CS and A-CS but there was a significant difference in UFC and cortisol serum levels in patients with ectopic source of CS vs. patients with P-CS and A-CS.

The most common feature of bone status was osteoporosis of spine (31.2%). The prevalence of fractures was 25% (the most frequent in E-CS group: 42.9%). Pecori Giraldi reported a prevalence of osteoporosis ranging from 31.6 to 46.8% in a gender-comparative study that involved 280 CS patients [8]. Ohmori et al. confirmed the prevalence of osteoporosis in 54.8% of CS subjects and fractures in 21.4% of them [51]. The prevalence of osteoporosis and fractures was lower in pituitary CS than in adrenal CS (69.6% vs. 37.8% and 26.1% vs. 15.8%, respectively). Other studies showed that ectopic etiology of CS may be associated with higher prevalence of vertebral osteoporosis [52, 53]. In our study, the prevalence of fractures was higher in E-CS group (42.9%) than in P-CS (24.4%) and A-CS (18.7%) groups.

Similarly to other studies on CS, the main limitation of our study was a small sample size. The most important limitation of the study is the fact that a small, heterogeneous group of patients with E-CS is compared to patients with P-CS and A-CS. Nonetheless, the low prevalence of CS should not discourage authors from collecting clinical data to analyze and compare epidemiological, biochemical and clinical features of pituitary, adrenal and ectopic CS. In accordance with the Endocrine Society Clinical Practice Guidelines, we evaluated retrospectively a group of patients from a single endocrinological center in order to collect more data on the clinical usefulness to develop diagnostic prediction model of features based on clinical signs and symptoms technically easy to obtain in clinical practice.

## Conclusions

This study of CS revealed significant differences in clinical presentation between ectopic, pituitary, and adrenal CS. The epidemiology of CS is changing toward the growing percentage of A-CS, probably associated with increasing detection of cortisol producing adrenal incidentalomas. Skin alterations (82.8%), weight gain (81.2%), and myopathy (78.1%) were predominant signs; hypertension (81.2%), dyslipidemia (68.7%) were the most common symptoms in all cases in our study. Our patients with E-CS had various metabolic complications (such as DM, dyslipidemia, hypertension) due to hypercortisolemia; their incidence is comparable to that from other studies. It is significant that all patients with E-CS in our study presented a profound hypokalemia. Salient hypokalemia could be a biochemical marker more suggestive for E-CS rather than P-CS. The incidence of diabetes is more frequent in E-CS group than in P-CS and A-CS groups.

Our study indicated that patients with E-CS often present with pronounced life-threatening disorders of glucocorticosteroid excess and significantly higher levels of UFC, cortisol serum, and ACTH. The clinical presentation of CS is related to the degree of cortisol excess and the disease duration. CS has to be diagnosed and treated as early as possible; short period of time from the onset of first symptoms to diagnosis is crucial to reduce mortality and morbidity. Physicians of all specializations, especially diabetologists, should be aware of secondary causes of DM, unusual comorbidities for age, particularly in the presence of profound hypokalemia, secondary hypertension and osteoporosis. These data have proved a long-time delay from the onset of first clinical features of CS to diagnosis of P-CS and A-CS. That is why we suggest that general practitioners and other specialists should search for the presence of rare or atypical signs and symptoms, such as striae rubra, facial plethora, proximal myopathy, easy bruising and unusual for age comorbidities, including DM, hypertension and osteoporosis, especially in patients with hypokalemia and weight gain.

## List of abbreviations

CS: Cushing’s syndrome
P-CS: pituitary dependent CS
A-CS: adrenal dependent CS
E-CS: CS from an ectopic source
UFC: urinary free cortisol
CDST: cortisol level after overnight 1 mg dexamethasone suppression test
ACTH: adrenocorticotropic hormone
BMI: body mass index
DM: diabetes mellitus
IFG: impaired fasting glucose
SP: systolic blood pressure
DP: diastolic blood pressure
LDDST: low dose dexamethasone suppression test
